# Gastric and Duodenal Pseudomelanosis: An Extended Unusual Finding in a Patient with End Stage Kidney Disease

**DOI:** 10.1155/2016/2861086

**Published:** 2016-03-02

**Authors:** Noor Ul Ain Qureshi, Muhammad Faraz Younus, Kourosh Alavi, Muhammad Yasin Sheikh

**Affiliations:** ^1^Division of Gastroenterology and Hepatology, University of California San Francisco (UCSF), 1st Floor, Endoscopy Suite, 2823 Fresno Street, Fresno, CA 93721, USA; ^2^University of Nevada, 1155 Mill Street, Reno, NV 89502, USA

## Abstract

Gastric and duodenal pseudomelanosis is a rare endoscopic mucosal finding, characterized by the accumulation of iron in macrophages of the lamina propria of the stomach and duodenum. The clinical significance and long term sequelae have not been clarified yet. However, this benign condition is associated with a variety of clinical conditions, such as essential hypertension, chronic renal failure, diabetes mellitus, long term intake of iron supplements, and furosemide. Duodenal pseudomelanosis appears to be more common than gastric pseudomelanosis given the fact that a few cases of gastric pseudomelanosis have been reported in the literature so far. We report a case of 88-year-old lady with ESRD who is maintained on hemodialysis and presented with abdominal pain. An upper GI endoscopy showed discoloration of the antrum of the stomach and most portion of her duodenum. Histopathology report confirmed the presence of iron laden macrophages in the lamina propria of both stomach and duodenum.

## 1. Introduction

Gastric and duodenal pseudomelanosis (GDP) is a benign and infrequent condition of uncertain clinical significance and it is associated with a variety of clinical conditions and intake of several medications [1, 2]. The condition was first described in 1976 by Bisordi and Kleinman [3]. Diagnosis of GDP is based on an endoscopic finding of the stomach and duodenum, which shows dark pigmentation of the stomach and duodenal mucosa because of deposition of pigment in lamina propria macrophages. Although the pigment was mostly found to consist of ferrous sulfate, small amounts of other elements were also found [4]. The majority of diagnosed patients belong to the adult population with female predominance [1], but a case of duodenal pseudomelanosis without gastric pseudomelanosis in adolescence male has also been published in the literature [5]. Long term prognosis and sequelae of this condition is yet to be determined.

## 2. Case Report

**Figure 1:**
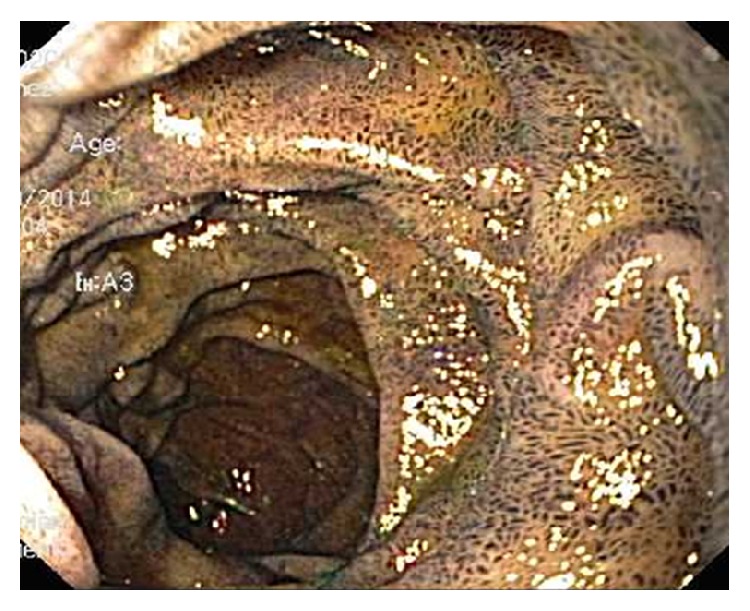
Endoscopic examination of duodenum showing dark speckled appearance of duodenal mucosa.

**Figure 2:**
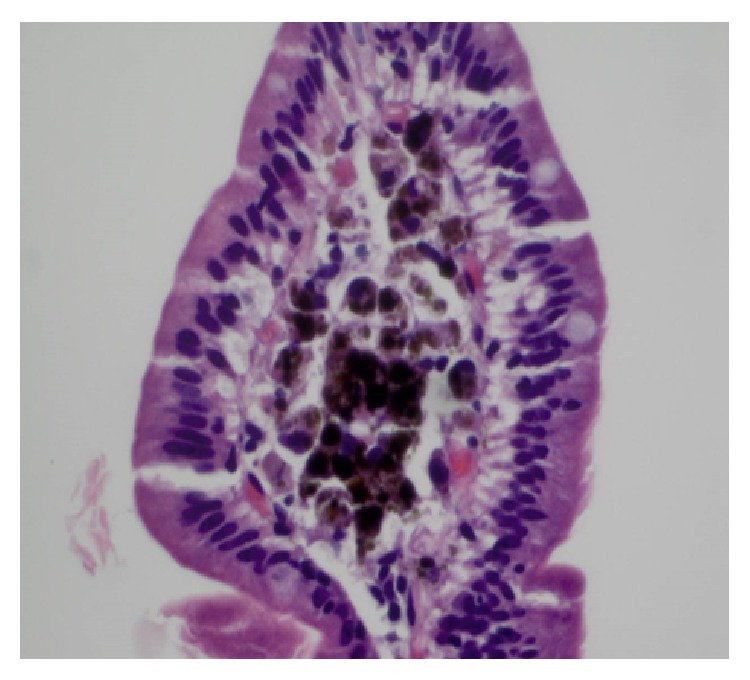
Histological examination of duodenal biopsy showing black pigmentation.

**Figure 3:**
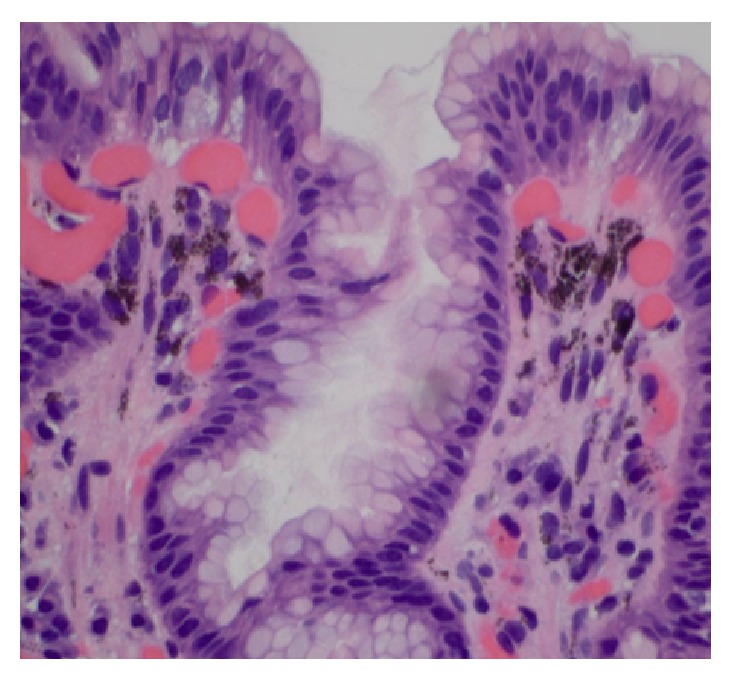
Image 60x of Lamina propria of stomach showing deposition of black pigmentation.

This 88-year-old lady with ESRD on hemodialysis, anemia, GERD, hyperlipidemia, COPD, and CHD was admitted to our hospital with complaints of abdominal pain, odynophagia, and vomiting for 4 days. Pain was diffused and dull in nature with no radiation. Vomitus was nonbilious and nonbloody. She reported similar episodes over the last three months. She has been taking ferrous sulfate for her anemia, for the last one year. Previous endoscopy, which was done two years back, was unremarkable, although endoscopy, which was done three months back, showed discoloration of mucosa of duodenum without any discoloration of the stomach mucosa. Upper endoscopy at this time revealed scattered moderate mucosal abnormality characterized by discoloration of the antrum and part of her corpus of the stomach. No ulcers, erosions, or any other causes for bleeding were identified. Also patchy mild inflammation characterized by erosion, erythema, and friability was found on the lesser curvature of the stomach. A diffuse moderate mucosal abnormality of the duodenum characterized by discoloration was found in the entire duodenum extending to the fourth part (Figure 1). Histopathology report confirmed the presence of loose aggregates of macrophages containing a granular black pigment within the superficial lamina propria (Figures 2 and 3). Iron stain showed a portion of this pigmented material to be composed of iron.* Helicobacter pylori* organism was absent. Therefore, the diagnosis of gastric pseudomelanosis and duodenal pseudomelanosis was confirmed. The iron therapy was discontinued and subsequently patient did fairly well.

## 3. Discussion

Although etiology and clinical significance of this condition have not been established yet, GDP is associated with a variety of clinical conditions including hypertension, hemochromatosis, diabetes, chronic renal disease, gastrointestinal bleeding, and intake of several medications including iron sulfate and certain antihypertensive medication such as furosemide, hydralazine, propranolol, and hydrochlorothiazide [2, 5]. Clinically this process has been documented in adults, more commonly in older women.

It has been suggested that such pigmentation may cause problems with intestinal absorption of nutrients [6, 7]. Although the diagnosis of GDP is clear on endoscopy nevertheless differential diagnosis includes necrosis, metastatic melanoma, and ingestion of charcoal [8], brown bowel syndrome, hemosiderosis/hemochromatosis [9], and alkalis [10]. GDP is mostly asymptomatic but cases have been reported in the literature in which patients presented with dyspeptic symptoms.

Histologically, pigments are deposited in the lysosomes of macrophages in the lamina propria [11]. The pigments mostly consist of iron, but small amounts of calcium, aluminum, potassium, magnesium, sulfur, silicon, and silver have also been detected. The electron microscope usually shows membrane bound, electron dense, and smooth to crystalline bodies. It has been suggested that these trace elements are probably derived from the biological milieu and/or other drugs and/or food contamination [11].

The predominant pigments deposited are iron oxide and hemosiderin. These pigments are detected by both the Prussian blue and Fontana-Masson stains of macrophages in the lamina propria. It has been suggested that in duodenal pseudomelanosis iron metabolism may be impaired and iron is pooled within macrophages [12]. One case report also suggests that pigmentation can occur in the absence of a history of oral iron supplementation [13]. In such cases, pigments may be derived from hemorrhage in the gastrointestinal tract. Antihypertensive drugs may have a role in the causation of the pigmentation formation.

Long term prognosis and sequelae of this condition are not known and no specific protocol for treatment and follow-up has not determined yet.

## 4. Conclusion

Gastric along with duodenal pseudomelanosis is a very rare, benign condition of unknown clinical significance. This condition is asymptomatic per se, but the patient may present with dyspeptic symptom. In such cases, it is important to exclude other causes of dyspepsia by endoscopic and laboratory evaluation. Proper knowledge and understanding of the common associations of this condition may help in avoiding unnecessary workup. The longer duration of iron therapy in the setting of ESRD might be responsible for extensive pigmentation involving both stomach and duodenum in our case. However, further studies are needed to prove this association.
